# Fatty acid patterns early after premature birth, simultaneously analysed in mothers' food, breast milk and serum phospholipids of mothers and infants

**DOI:** 10.1186/1476-511X-8-20

**Published:** 2009-06-10

**Authors:** Karl-Göran Sabel, Cristina Lundqvist-Persson, Elsa Bona, Max Petzold, Birgitta Strandvik

**Affiliations:** 1 Department of Paediatrics, Borås Hospital, Borås, Sweden; 2 Skaraborg Institute, Skövde, Sweden; 3 The Nordic School of Public Health, Göteborg, Sweden; 4 The Department of Paediatrics, the Sahlgrenska Academy, Göteborg University Göteborg, Sweden

## Abstract

**Background:**

The supply of long-chain polyunsaturated fatty acids via the placenta is interrupted in premature infants, making them exclusively dependent on breast milk, which varies in fatty acid (FA) concentrations depending on the mother's diet.

**Objective:**

To in a longitudinal study explore the relation between FA status in mothers and infants from an unselected cohort of prematures, not requiring intensive care.

**Design:**

Breast milk and mothers' and infants' plasma phospholipid FA concentrations from birth to 44 weeks of gestational age were analysed and compared with mothers' food intake, assessed using a 3-day diary. Fatty acids were analysed by capillary gas-liquid chromatography.

**Results:**

The energy intake was low in 75% of mothers, and 90% had low intake of essential FAs (EFAs). Dietary linoleic acid (LA, 18:2w6), but not w3 FAs, correlated to concentrations in breast milk. Infants' plasma and breast milk correlated for arachidonic (AA, 20:4w6), eicosapentaenoic (EPA, 20:5w3) and docosahexaenoic (DHA, 22:6w3) acids. A high concentration of mead acid (20:3w9) in the infants at birth correlated negatively to the concentrations of LA, AA and w3 FAs. Infants of mothers who stopped breastfeeding during the study period showed decreased DHA concentrations and increased w6/w3 ratios, with the opposite FA pattern seen in the mothers' plasma.

**Conclusion:**

Although dietary w3 FAs were insufficient in an unselected cohort of mothers of premature infants, breastfeeding resulted in increased levels of DHA in the premature infants at the expense of the mothers, suggesting a general need to increase dietary w3 FAs during pregnancy and lactation.

## Introduction

The importance of lipids for growth and development of the central nervous system (CNS) was addressed by Widdowson in 1968 [1], but has been given more attention during the latest decades [2-4]. During the last trimester of pregnancy, a substantial number of essential fatty acids (EFAs), predominantly the long-chain polyunsaturated fatty acids (LCPUFAs) are transferred from the mother to the foetus [5,6]. During this time and in the early neonatal period, rapid synthesis of brain tissue, with cellular differentiation and active synaptogenesis, has a special need for docosahexaenoic (DHA) (22:6w3), eicosapentaenoic (EPA) (20:5w3) and arachidonic (AA) (20:4w6) acids. From 26 weeks of gestation until term 80% of the brain DHA accrues in the foetus [3], and recent research has also stressed the importance of DHA for normal development of the glial cell [7,8]. This challenge is met by increased extraction of LCPUFAs via the placenta, and also by some synthesis from the precursors linoleic acid (LA) (18:2w6) and α-linolenic acid (ALA) (18:3w3) [9,10]. The LCPUFAs transferred from mother to foetus in the last trimester and during the early weeks of life have been calculated to be 50 mg/kg/day for w3 and 400 mg/kg/day for w6 FAs [11].

In premature infants this supply is interrupted and the infant is exclusively dependent on breast milk, which varies regarding FA content, mainly depending on the mother's diet [12-14]. Human breast milk contains EFAs of the w6 and the w3 series, while until recently most formulas have not been supplemented with LCPUFAs [15].

Breast milk is generally considered the gold standard of infant feeding despite well-documented variations related to the mother's diet and her lipid stores [9,16-18], and it is not clear whether the extended requirements of the premature infant are met. Some authors report a higher concentration of LCPUFAs in the milk of mothers giving birth to premature infants [19]; others report no difference from those giving birth to full-term infants [20-22].

Premature birth has also been discussed as a mode of programming for later diseases and has been compared to programming due to undernutrition [23]. Disturbances in FA metabolism may have long-term consequences because FAs influence gene expression [24]. According to Raju [25], late-preterm infants present many clinical problems, which are reflected in higher rates of rehospitalization. The incidence of severe sequelae after preterm birth has not decreased in recent years [26]. The long-term problems found also in apparently healthy late premature infants [25,27,28] have raised the question whether differences in early development may be related to variations in polyunsaturated fatty acids (PUFAs) in breast milk, depending on the maternal diet. Changes in the Western diet [14,29] and the increased demand for w3 FAs during the perinatal period [3] may be a potential risk for suboptimal development of infants born preterm. Most previous studies have focused on either mothers' or infants' FA concentration in plasma, often investigating infants fed formula with LCPUFA and using the breastfed infants as controls.

We therefore performed an explorative study of an unselected sample of breastfed preterm infants to investigate whether it is justified to always consider breastfed infants as controls, i.e. as gold standard. The included infants were without severe morbidity requiring neonatal intensive care, and their mothers were investigated with respect to food intake and the concentration of FAs in breast milk at 1 week after birth, as well as in the phospholipids of plasma of mothers and infants at the same time, and at ages comparable to the gestational ages of 40 and 44 weeks. This part of the investigation will address the FA composition in infants' compared with their mothers'plasma and in relation to the mothers' diet. Short- and long-term prospective studies, including motor and mental development, will be reported separately.

## Subjects and methods

### Subjects

Fifty-one newborn infants born prematurely after 24–36 completed weeks of gestation were consecutively included after permission from their parents. Infants with congenital malformations or whose mothers did not speak Swedish were excluded. The study comprised 23 boys and 28 girls, including eleven pairs of twins. The distribution of gestational age and birth weight is given in Table 1. Six infants were small for gestational age [30]. Mean (± standard deviation, SD) birth length was 44.5 (3.4) cm, knee-heel distance 110.3 (9.8) mm and head circumference 31.0 (2.2) cm. Infant morbidity, including asphyxia, mechanical ventilation for > 10 minutes, hypoglycaemia persisting > 6 hours, convulsions, and septicaemia or other infections, was present in 19 infants (37%). Basic data on the mothers are given in Table 2. Morbidity among mothers was defined as any chronic disease or complication related to pregnancy, and was noted in 17 mothers (43%). Complicated delivery ending in vacuum extraction or Caesarean section was reported in 21 mothers. Eight mothers were cigarette smokers.

**Table 1:** Distribution and mean (SD) of gestational age and birth weight and Apgar score in 51 preterm infants.

Gestational age, weeks	33.1 (2.6)
	n (%)
24–28 wks	3 (5)
28–32 wks	10 (20)
32–36 wks	38 (75)
	
Birth weight, kg	2.01 (0.59)
	n (%)
	
< 1,000 g	2 (4)
1 000–1 500 g	10 (20)
1 501–2 000 g	12 (23)
2 001–2 500 g	14 (27)
2 501–3 000 g	12 (23)
> 3 000 g	1 (2)
	
Apgar score at 5 min	n (%)
1–3	2 (4)
4–6	5 (10)
7–10	44 (86)

**Table 2:** Distribution and mean (SD) of age, parity and body mass index (BMI) of the mothers (n = 40).

Age of mother (yrs)	29.8 (6.0)
	n (%)
< 20 yrs	2 (5)
20–30 yrs	24 (60)
31–40 yrs	12 (30)
> 40 yrs	2 (5)
	
Parity	n (%)
1st	19 (48)
2^nd^	15 (37)
3^rd^–4^th^	6 (15)
	
BMI (kg/m^2)^	24.9 (3.9)
	n (%)
19–20.9	4 (10)
21–24.9	15 (38)
25–29.9	15 (38)
30–36	5 (13)

The body mass index (BMI) (kg/m^2^) of the mothers was calculated from weight and height measured at a time corresponding to 44 weeks of gestation.

### Food assessments

One week (6–10 days) after delivery the mother's food intake was assessed using a food diary. Following detailed instructions from the dietician the dietary intake was recorded during 3 consecutive days including 1 weekend day. Food content was calculated by the dietician using a nutrition registration program (Kostplan Dietist version 2.40, Aivo AB, Sweden) and related to the Nordic Nutrition Recommendations (NNRs) of 2004 [31]. After 6 months the reliability of the food assessment was tested in 25 of the 40 mothers.

### Collection of samples, and lipid analyses

#### Breast milk

At the time of dietary recording, i.e. about 1 week after delivery, the mothers' milk was collected over 24 hours using an electric pump. The 24-hour sample was kept at +4°C during collection, carefully blended and an aliquot of 5–10 mL was frozen at -70°C until analysis of FA patterns of the total lipids within 4 weeks. One hundred μL of milk was lyophilized overnight and lipids were extracted three times using sonication and centrifugation at 2 000 g. Butylated hydroxytoluene (BHT) was added (0.1 mg/mL chloroform) and the supernatant was evaporated under nitrogen and redissolved in 3 mL chloroform before methylation, as previously described [32].

#### Cord blood and plasma samples

Cord blood was obtained from 28 premature infants, but only 14 of these completed the study. Blood samples were collected from mothers and infants approximately 1 week after delivery (range 5–12 days, "early samples"), and again when the infants had reached the ages corresponding to 40 and 44 weeks of gestation. After centrifugation the plasma was frozen at -70°C until analysis of plasma phospholipids. During this procedure ten plasma samples of all those collected later were discarded accidentally.

#### Fatty acid analysis

Fatty acids were analysed as previously described [32]. After lipid extraction the plasma phospholipids were fractionated on a single SEP-PAK aminopropyl cartridge (Waters Corp., Milford, MA, USA) and eluated with methanol after washing with chloroform:isopropanol 2:1 and 2% HAc in ether. The fraction of lipids was transmethylated in methanolic-HCL-3N at 80°C over 4 hours. The FA methyl esters were separated by capillary gas-liquid chromatography in a Hewlett-Packard 6890 gas chromatograph. Heneicosanoic acid (21:0) was used as internal standard and the FA methyl esters identified by comparison with retention times of pure reference substances (Sigma Aldrich Sweden AB, Stockholm, Sweden). The coefficients of variation were 1.7% for LA, 2.9% for AA and 4.3% for DHA (n = 24).

The ratio between eicosatrienoic (mead) acid (20:3w9) and AA, i.e. the EFA deficiency index, was used as a marker of EFA deficiency [33].

### Ethics

The study was approved by the Ethics Committee of the Medical Faculty (the Sahlgrenska Academy) of the University of Göteborg, Göteborg, Sweden. Informed consent was obtained from all mothers.

### Statistical analysis

This is an exploratory study of associations between different variables. The associations have been assessed as correlations for continuous variables and mean differences between categories, and via regression analysis. These estimates have been used to indicate the size of the associations. Statistical significances (p-values) have been used to further assess the likelihood of a true association. However, because of the explorative nature of the paper, working without pre-specified hypotheses, no adjustment for multiple testing has been done. P-values are reported and statistical significance is stated for p < 0.05. The sample consists partly of twins, which is important to recognize when estimating the standard errors and calculating the significances, therefore key results were analyzed using both multilevel modelling and ordinary regression. No major differences were found and ordinary regression was then used for simplicity throughout the analysis. Means (± standard deviation (SD)) or confidence intervals (CI) or medians (range) are given. Multiple regressions were constructed by the stepwise enter method based on the significances in the simple regressions. Mean differences between groups were calculated from independent or paired t-tests where appropriate, otherwise by non-parametric tests. Pearson's correlation test was employed. Statistical calculations were computed using SPSS 14.0 (SPSS Inc., Chicago, IL, USA).

## Results

### Mothers' dietary intake at 1 week

Mothers of singletons (n = 29) were compared with mothers of twins (n = 11) using independent-samples *t*-test. There were no significant differences in nutritional data between the two groups.

The mean total energy intake was lower than the generally recommended intake levels for healthy women during the third trimester (Table 3). Thirteen mothers had an energy intake < 2 000 kcal/day, and in nine mothers the energy intake exceeded the recommended levels. The percentage fat energy intake (E%) was high in 20% of the mothers; but in five mothers the intake was < 25 E%. The ratio of polyunsaturated to saturated fatty acids (P/S ratio) showed a wide range with a negatively skewed distribution. The intake of monounsaturated fatty acids (MUFAs) was below the recommended level in 17 (42%) of the mothers, and in grams it was closely correlated to total fat intake (r = 0.95, p < 0.0001). The dietary content of EFAs (w6+w3 FAs) was low and was correlated to total fat intake (E%) (r = 0.68, p < 0.0001); in 13 mothers (33%) it was < 3 E%, and in 35 (88%) < 5 E%; none had an intake ≥ 10 E%. Five mothers consumed < 2 E% LAs. Significant correlations were found between E% of total fat and LA (r = 0.66, p < 0.0001), and w3 FAs (r = 0.69, p < 0.0001). The intake of w3 PUFAs constituted < 0.5 E% in twelve mothers (30%), while 36 (90%) consumed < 1.0 E%. A very low w3 intake (< 0.5 E%) was combined with a low total EFA content in the diet (< 3 E%) in 11/40 mothers. The mean dietary intake (95% CI) of ALA was 1.47 g/d (1.25–1.69), and of EPA+DHA 0.20 g/d (0.13–0.26). Also, the LA/ALA ratio, as well as the w6/w3 FA ratio, varied without extremes, and as expected the correlation between these two ratios was high (r = 0.82, p < 0.0001). Finally, the intake of vitamin E was 7.7 mg/d (6.4–9.1); vitamin C 127 mg/d (106–148); Fe 11.0 mg/d (9.7–12.2) and Zn 11.6 mg/d (10.7–12.5), all within the recommended levels [31] (data not shown).

**Table 3:** Composition of macronutrients in mothers' self-reported dietary intake.

Variable	Range	Mean (95% CI)	NNR 2004
Energy, kcal	1382 – 3153	2266 (2119 – 2415)	2700 kcal
Carbohydrate, E%	38.3 – 74.1	55 (52.5 – 57)	55 – 60 E%
Sucrose, E%	2.8 – 22.7	12.5 (11 – 14)	< 10 E%
Protein, E%	10.7 – 20.1	15 (14 – 15)	10 – 20 E%
Fat, E%	12.4 – 45.5	30 (28 – 32)	25–35 E%
SFA, E%	4.4 – 19.6	14 (13 – 14)	10 E%
Trans fat, E%	0.15 – 1.85	0.9(0.75 – 1.0)	Incl in SFA
P/S ratio	0.15 – 0.63:1	0.3 (0.25 – 0.3):1	0.14 – 0.20:1
MUFA, E%	4.0 – 16.8	10 (10 – 11)	10 – 15 E%
PUFA, E%	1.7 – 7.5	4 (3 – 4)	5 E%
LA, E%	1.4 – 6.4	3 (2.5 – 3)	1.5 -4 E%
EFA w3, E%	0.3 – 1.3	0.7 (0.6 – 0.75)	1 E%
Ratio LA/ALA	2.3 – 7.5:1	5 (5 – 5.5):1	4:1
Ratio w6/w3	2.3 – 7.0:1	5 (4 – 5):1	4–5:1

A mean of 3 days, compared with the Nordic Nutrition Recommendations (NNR 2004). SFA = saturated fatty acids, MUFA = monounsaturated fatty acids, PUFA = polyunsaturated fatty acids, E%, percent energy of total energy in food, LA = linoleic acid, EFA = essential fatty acids, ALA = α-linolenic acid.

Twenty-five of the mothers agreed to repeat a 3-day food assessment performed at home about 6 months after the first assessment. In twelve of the 13 variables there were no differences between the two investigations. The only exception was a lower energy intake with a mean (± SD) of 1 790 (400) compared with 2 250 (400) kcal/day at the first assessment (p < 0.0001).

### Breast milk at 1 week

The premature infants were given early feeds of breast milk from the milk bank within 1–2 hours of birth. These were exchanged gradually with the mothers' own milk within 3.0 (1.0) days. Thirty-six infants were exclusively fed breast milk for between 2 weeks and 8.5 months (median 3.0 months), while partial breastfeeding was utilized in 15 infants for 1–14.5 months (median 3.0 months). Breast milk substitutes used were LCPUFA-enriched Pre-Nan (Nestlé, Helsingborg, Sweden) and in the older premature infants Baby-Semp 1 (Semper AB, Stockholm, Sweden).

Twenty-eight milk samples were from mothers of singletons and eleven were from mothers of twins, corresponding to the milk given to 50 infants (Table 4). The milk from mothers of singletons was no different from that of mothers of twins with the exception of palmitoleic acid (16:1w7), which was lower in milk from mothers of twins (p = 0.027). Trans FAs, reported as the sum of 16:1w7T and 18:1w9T, did not exceed 4.2 mol% (mean 2.3 (1.1) mol%). There was no significant correlation between LA and AA, or between ALA and DHA, while EPA and DHA were closely associated (r = 0.74, p < 0.0001). Mead acid and AA were positively associated in breast milk (r = 0.44, p = 0.006, n = 39).

**Table 4:** Composition of fatty acids (mol %) in cord blood, mothers'and infants' plasmaphospholipids and breast milk at 1 week postnatally.

Fatty acid	Cord plasma	Mothers' milk	Mothers' plasma	Infants' plasma	Correlations, r (p)	
	Mean (SD) (n = 28)	Mean (SD) (n = 39)	Mean (SD) (n = 30)	Mean (SD) (n = 39)	mothers'milk – mo plasma (n = 30)	milk – infant early plasma (n = 39)

12:0	0.004 (0.01)	4.7 (2.5)§§	0.01 (0.01)**	0.03 (0.03)¤		
14:0	0.44 (0.09)	8.2 (3.0)§§	0.52 (0.13)**	0.42 (0.13)¤¤	0.58 (0.001)	0.36 (0.03)
16:0	35.6 (1.5)⊗⊗	27.4 (2.1)§§	34.1 (1.3)**	31.5 (1.7)¤¤		
18:0	13.3 (1.6)	6.8 (1.0)§§	10.8 (1.2)**	13.8 (1.5)¤¤		
Σ SFA	53.3 (1.0)⊗⊗	48.0 (5.7)	48.7 (0.9)	48.8 (1.3)	0.34 (0.07)	
						
16:1w7	1.2 (0.3)⊗	2.74 (0.59)§§	0.72 (0.21)**	0.74 (0.29)	0.42 (0.02)	
18:1w9	8.4 (1.4)⊗⊗	35.9 (4.1)§§	10.0 (1.2)**	11.7 (1.1)¤¤		
24:1w9	3.3 (0.4)	0.36 (0.23)§§	3.4 (0.6)**	3.3 (0.5)		
Σ MUFA	12.9 (1.9)⊗⊗	39.3 (4.2)§§	14.1 (1.5)**	15.7 (1.6)¤¤		
						
20:3w9 Mead	0.67 (0.32)	0.04 (0.02)§§	0.25 (0.12)**	0.49 (0.27)¤¤		
						
18:2w6 (LA)	8.1 (1.5)⊗⊗	9.1 (1.9)§§	19.0 (2.3)**	15.7 (2.0)¤¤	0.35 (0.06)	
18:3w6 (GLA)	0.06 (0.03)	0.05 (0.05)	0.05 (0.03)	0.07 (0.05)¤		
20:3w6 (DGLA)	5.0 (0.8)⊗⊗	0.43 (0.12)§§	3.6 (0.8)**	3.5 (0.5)	0.70 (0.000)	0.36 (0.02)
20:4w6 (AA)	14.4 (2.0)⊗	0.53 (0.09)§§	8.0 (1.2)**	11.3 (1.6)¤¤	0.41 (0.03)	0.46 (0.004)
Σ w6 FA	27.8 (2.0)⊗	10.5 (2.0)§§	30.8 (1.6)**	31.0 (2.1)	0.36 (0.05)	
						
18:3w3 (ALA)	0.05 (0.01)⊗⊗	1.31 (0.40)§§	0.35 (0.10)**	0.11 (0.03)¤¤		
20:5w3 (EPA)	0.48 (0.21)	0.09 (0.03)§§	1.07 (0.38)**	0.66 (0.19)¤¤	0.58 (0.001)	0.68 (0.000)
22:6w3 (DHA)	4.8 (1.3)⊗	0.45 (0.15)§§	4.34 (1.00)**	3.3 (0.52)¤¤	0.84 (0.000)	0.48 (0.002)
Σ w3 FA	5.3 (1.2)⊗	1.9 (0.46)§§	5.8 (1.15)**	4.1 (0.57)¤¤	0.42 (0.02)	
						
Σ PUFA	33.9 (2.1)	12.5 (2.33)§§	40.1(1.49)**	35.5 (2.08)¤¤	0.54 (0.002)	
						
Ratios:						
AA/DHA	3.1 (0.7)	1.3 (0.3)§§	1.9 (0.5)**	3.5 (0.6)¤¤	0.81 (0.000)	0.35 (0.03)
w6/w3	5.58 (1.14)⊗⊗	5.56 (0.94)§§	5.58 (1.29)	7.65 (1.00)¤¤	0.67 (0.000)	0.34 (0.03)

In mothers of twins only one value is used. Mean (SD).

The following comparisons were made by paired t-test:

⊗⊗ Comparison between cord plasma and early infant plasma: p < 0.000, and ⊗ p < 0.05, n = 14.

** Mothers' early plasma and breast milk: p < 0.001, n = 30.

§§ Infant early plasma and breast milk: p < 0.001, n = 39

¤¤ Mothers' plasma and infant plasma: p < 0.000, and ¤ p < 0.05, n = 38.

SFA = saturated fatty acids, MUFA = monounsaturated fatty acids, LA = linoleic acid, GLA = γ-linolenic acid, DGLA = dihomo-γ-linolenic acid, AA = arachidonic acid, ALA = α-linolenic acid, EPA = eicosapentaenoic acid, DHA = dococahexaenoic acid, PUFA = polyunsaturated fatty acids.

The P/S ratio in food was correlated to that in breast milk (r = 0.74, p < 0.0001), and to the ALA (r = 0.72, p < 0.0001) and LA (r = 0.62, p < 0.0001) concentrations in breast milk (n = 38). Significant correlations were also found between the dietary E% of LA and the concentration of LA in breast milk (r = 0.56, p < 0.0001, n = 38), while no correlations were found between w3 FAs, or the LA/ALA or w6/w3 FA ratios in the food intake and breast milk.

### Cord plasma

No significant difference in plasma phospholipid concentration of FAs was found between the cord blood from the 14 infants who completed the study and the group of 14 infants who did not. The two samples were therefore combined (Table 4). Negative correlations were found in the cord blood between palmitic (16:0) and stearic (18:0) acids (r = -0.75, p < 0.0001, n = 28), and between LA and AA (r = -0.55, p = 0.002, n = 28).

### Fatty acids in the mothers' plasma at 1 week

In 30 mothers, ten of whom had given birth to twins, blood samples for FA analyses were taken approximately 1 week after delivery (8.6 ± 2.3 days), at the same time as breast milk was collected.

The main w6 FAs, LA and AA (Table 4), were negatively correlated (r = -0.62, p < 0.0001, n = 30). In the w3 series, DHA predominated, followed by EPA, and a negative correlation was found between ALA and DHA (r = -0.41, p = 0.026, n = 30). The w6/w3 ratio varied from 3.4 to 9. Mead acid showed a negative correlation to LA, and a positive correlation to AA, while no correlations were found between mead acid and DHA or ALA in the early samples. In the multiple regression analysis, LA and AA were both significant contributors (β = -0.38, p = 0.032, and β = 0.42, p = 0.020, respectively; R = 0.72, R^2 ^= 0.52, p < 0.0001, n = 30), indicating that 52% of the variation of mead acid was explained by the concentration of these two FAs. Docosahexaenoic acid in mothers' plasma and in breast milk were strongly correlated (r = 0.82, p < 0.0001).

### Infants' plasma fatty acids at 1 week

**Figure 1:**
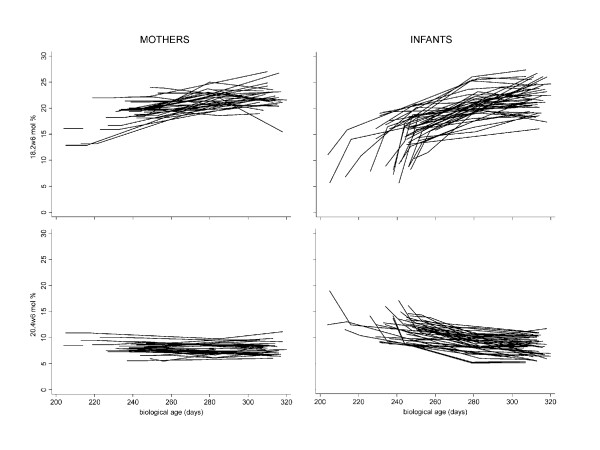
**Linoleic (18:2w6) and arachidonic (20:4w6) acid in serum phospholipids of mothers and their premature infants from 1 week after birth to 44 weeks of gestational age**.

**Figure 2:**
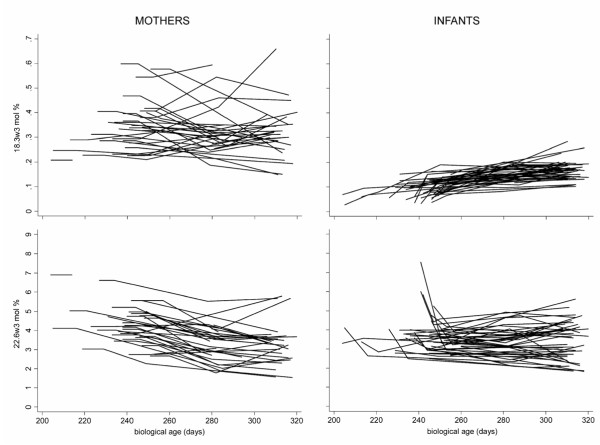
**Alpha-linolenic (18:3w3) and docosahexaenoic (22:6w3) acids in serum phospholipids of mothers and their premature infants from 1 week after birth to 44 weeks of gestational age**.

Blood samples were first drawn at a mean of 8.7 days after birth (at 5–11 days in 36 infants, on day 13 in one infant, and on day 16 in two infants). The samples from the three older infants were not outliers for any of the FAs, and were therefore included in the analyses (Table 4). Also the analyses of FAs in the 6 SGA infants did not differ and were therefore not separately analysed. As shown in Figures 1 and 2 the variation was large for w3 FA compared with w6 FA, especially in the mothers, and AA showed less individual variation than DHA in the infants, reflecting a strong correlation between DHA in mothers and infants. Similarly to cord blood, negative associations were found in infants' plasma between palmitic and stearic acids (r = -0.72, p < 0.0001), and between LA and AA (r = -0.36, p = 0.021) (n = 39). No correlation was found between ALA and DHA.

Multiple regression analysis with mead acid as dependent variable showed LA, AA and w3 FAs to be significant negative covariates (LA β = -0.61, p < 0.0001; AA β = -0.60, p < 0.0001; and w3 FA β = -0.26, p = 0.030; R = 0.78, R^2 ^= 0.60, p < 0.0001; n = 39), indicating that 40% of the variance of mead acid was explained by the concentrations of these three components in infants' plasma. Mead acid was independent of gestational age and birth size of the infant, but within 10–14 days of birth the plasma concentration had decreased markedly.

Some infants had a very low concentration of LA (< 14 mol%) in the early plasma sample (group 1, n = 9) and were compared with the rest of the group (group 0, n = 30). The infants with low LA levels more often had high AA (p < 0.05) and lower ALA (p = 0.001) concentrations, but the concentration of DHA was similar. The gestational age did not differ, but infants in group 1 were less growth retarded (-14% versus -24%, p = 0.001) and their birth was less complicated (p = 0.003). The mothers of group 1 had lower energy intake of total fat (p < 0.05) and MUFA (p = 0.006). In infants' early plasma samples, lower values were found in group 1 for all PUFA (p = 0.007), w6 FAs (p = 0.001) and for the w6/w3 ratio (p = 0.03). That group also had higher concentrations of palmitic (16:0) (p = 0.001), palmitoleic (16:1w7) (p = 0.002), behenic (22:0) (p = 0.03), lignoceric (24:0) (p = 0.003) and nervonic (24:1w9) (p = 0.02) acids, which is consistent with the finding that the sum of saturated FAs (SFA) in infants' early plasma was higher in group 1 (p = 0.009). Also, the oleic (18:1w9, OA)/LA ratio was higher (p = 0.001). All these findings indicated a higher endogenous fat synthesis, probably compensating for the lower fat E% intake in these mothers.

### Comparisons between fatty acids in cord blood, and the relations between fatty acids in breast milk and plasma phospholipids of mothers and infants sampled at the same time

Compared with infants' plasma phospholipid FA patterns at 1 week of age, the cord blood (n = 14) had 48% lower concentrations of LA, but higher concentrations of dihomo-γ-linolenic acid (DGLA, 20:3w6) and AA. The ALA concentrations were very low; while DHA was 45% higher in cord plasma than in infants' early plasma samples (Table 4).

Comparing cord plasma and mothers' plasma phospholipid FAs at 1 week postnatally (n = 13), the former had lower LA, but higher AA concentrations (p < 0.0001 for both). The concentrations of the w3 FAs ALA and EPA were lower in cord plasma than in the mothers' early plasma samples (p < 0.0001 and p = 0.001, respectively). Docosahexaenoic acid had a similar concentration in the two samples, explaining that the AA/DHA ratio was higher in cord plasma (p < 0.0001). Finally, mead acid was higher in cord plasma (p = 0.002) than in mothers' early plasma samples, but showed no correlation to either w6 or w3 FAs.

The FA composition of plasma phospholipids showed major similarities between mother and infant at 1 week of age although the difference between the samples was significant, especially for LA and the w3 FAs, which all showed higher concentrations in mothers' plasma. There were few significant correlations between mothers' and infants' plasma except for the w3 FAs ALA (r = 0.53, p = 0.001), EPA (r = 0.49, p = 0.002) and DHA (r = 0.37, p = 0.022) (n = 38).

**Figure 3:**
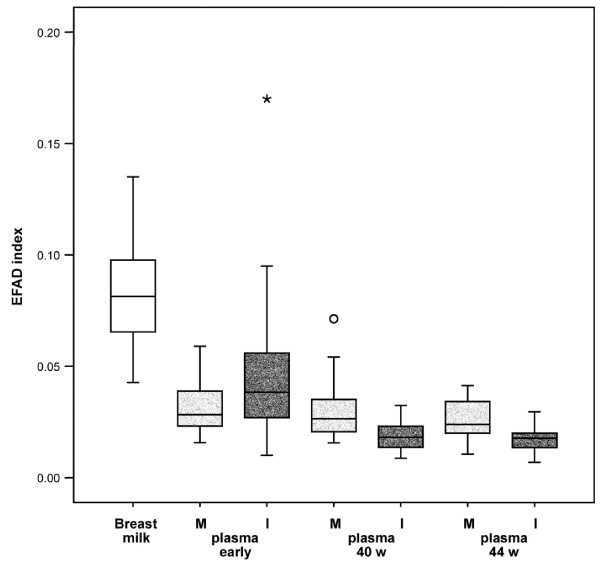
**Box plot of the essential fatty acid (EFA) deficiency index (the mead acid 20:3w9/arachidonic acid 20:4w6 ratio) in breast milk (unfilled) and in mothers' (M, grey) and infants' (I, dotted) plasma phospholipids from 1 week of age (early) to 44 weeks of gestational age**. The boxes indicate medians and 25^th^–75^th ^percentiles. Whiskers indicate 5^th ^to 95^th ^percentiles and outliers are indicated by separate symbols.

The composition of breast milk is the major determinant of the infant's plasma FA concentrations after birth, and we therefore compared the different compartments although the difference in their type has to be considered and consequently the results need to be evaluated with caution. The total concentration of SFA in breast milk was similar to that of mothers' plasma phospholipids (Table 4), but with higher concentrations of lauric (12:0) and myristic (14:0) acids and lower concentration of palmitic and stearic acids, also in relation to the infants' plasma phospholipids analysed at the same time. The biological significance of the high amount of these shorter FAs in breast milk has not been explored. There was a marked increase in OA and palmitoleic (16:1w7) acids, but not in nervonic acid (24:1w9) in mothers' milk compared with infants' plasma. The ratio of OA/LA was also substantially higher in mothers' milk than in plasma (p < 0.0001). The concentrations of the w6 FA LA, and especially DGLA and AA, were substantially lower (total about 66%) than in mothers' and infants' plasma phospholipids. The w3 FAs likewise showed similar lower concentrations in mothers' milk than in plasma, except for ALA, which was three times and twelve times higher than in mothers' and infants' plasma, respectively. A close correlation was found between DHA as well as EPA in mothers' plasma and milk (Table 4). Mead acid was lower in breast milk compared with mothers' plasma, while the EFA deficiency index was higher (Figure 3). A significant correlation was found between AA in mothers' milk and infants' plasma and between EPA and DHA in mothers' milk and infants' plasma, but not for LA or ALA (Table 4).

In order to investigate whether some factors would be identified to predict the concentration of w6 and w3 FAs in mothers' milk, and in infants' early plasma samples, the concentrations of w6 and w3 in breast milk were tested for possible correlations with background factors, viz. mothers' food and mothers' plasma. The w6 FAs in breast milk were best predicted by the P/S ratio in food, and the PUFA concentration in mothers' plasma (β = 0.58, p < 0.0001, and β = 0.31, p = 0.039, respectively; R = 0.76, R^2 ^= 0.58, p < 0.0001, n = 29). Seventy-two per cent of the w3 FA concentration in breast milk was explained by three factors, namely the P/S ratio in mothers' food (β = 0.66, p < 0.0001), the sum of w3 FAs in mothers' plasma at the same time (β = 0.42, p = 0.001), and gestational age (days) (β = 0.28, p = 0.016; R = 0.85, R^2 ^= 0.72, p < 0.0001, n = 29). No PUFA in mothers' diet correlated significantly to predict w6 in infants' plasma from background factors, mothers' plasma and breast milk. On the other hand, the w3 FA concentration in infants' plasma was predicted by two factors: DHA in breast milk (β = 0.51, p < 0.0001) and the OA/LA ratio in mothers' plasma (β = -0.28, p = 0.045; R = 0.63, R^2 ^= 0.40, p < 0.0001, n = 38). The analyses therefore indicated that the P/S ratio in mothers' food intake and their intake of w3 FAs accounted for about 40% of the variance of EFAs in infants' early plasma samples.

### Longitudinal study of mothers' plasma fatty acids up to 44 weeks

Comparisons of mothers' early plasma sample and the samples of infants corresponding to a gestational age of 40 weeks showed increases in LA (p < 0.0001) and the AA/DHA ratio (p < 0.0001, n = 31). Docosahexaenoic acid levels decreased with time (p < 0.0001, n = 31), but showed great individual variation, as did the ALA concentration (Figure 2). Also, the w6/w3 and LA/ALA ratios increased significantly between the early samples and those corresponding to the gestational age of 44 weeks (n = 32, p < 0.0001 for both). No difference was detected in mothers' plasma between 40 and 44 weeks for any FA except EPA (Table 5). The variability of w3 FAs was much larger than that of w6 FAs in the mothers. Strong correlations were found for most FAs measured at 40 and 44 weeks. At 40 weeks, > 60% of the results for mead acid were explained by two negative covariates, LA and DHA, which was even more pronounced at 44 weeks (LA β = -0.67, p < 0.0001; DHA β = -0.63, p < 0.0001; R = 0.80, R^2 ^= 0.64, p < 0.0001, n = 27).

**Table 5:** Serum phospholipid fatty acids (mol %) in mothers' and infants' plasma at 40 and 44 week.

Fatty acid	Mothers' plasma at 40 w (n = 29)	Mothers' plasma at 44 w (n = 29)	r (p) mothers 40 and 44 w (n = 29)	Infants' plasma at 40 w (n = 36)	Infants' plasma at 44 w (n = 45)	r (p) infants 40 and 44 w (n = 36)
12:0	0.02 (0.01)	0.02 (0.01)	0.66 (0.000)	0.03 (0.02)	0.03 (0.02)	0.55 (0.000)
14:0	0.49 (0.13)	0.45 (0.11)	0.57 (0.001)	0.44 (0.11)	0.38 (0.10)§	0.52 (0.001)
16:0	32.1 (1.17)	31.6 (1.29)	0.61 (0.001)	28.5 (1.16)	28.9 (1.43)§	0.78 (0.000)
18:0	12.3 (1.19)	12.4 (0.91)	0.78 (0.000)	16.2 (1.14)	15.9 (1.21)§	0.73 (0.000)
Σ SFA	48.3 (0.69)	47.9 (1.10)	0.63 (0.000)	48.2 (0.77)	48.3 (0.89)	0.29 (0.140)
						
16:1w7	0.70 (0.19)	0.58 (0.16)	0.46 (0.012)	0.40 (0.08)	0.35 (0.08)§§	0.82 (0.000)
18:1w9	10.2 (1.17)	10.0 (0.99)	0.62 (0.000)	11.2 (0.75)	11.0 (0.94)	0.54 (0.001)
24:1w9	2.98 (0.40)	3.00 (0.42)	0.67 (0.000)	2.81 (0.49)	2.67 (0.47)§	0.50 (0.002)
Σ MUFA	13.8 (1.41)	13.6 (1.04)	0.75 (0.000)	14.4 (0.86)	14.0 (0.82)§	0.52 (0.001)
						
20:3w9 Mead	0.22 (0.09)	0.21 (0.09)	0.69 (0.000)	0.16 (0.06)	0.14 (0.04)§	0.75 (0.000)
						
18:2w6 (LA)	21.6 (1.47)	22.0 (2.29)	0.38 (0.045)	20.9 (2.47)	22.0 (2.74)§	0.68 (0.000)
18:3w6 (GLA)	0.08 (0.05)	0.08 (0.05)	0.68 (0.000)	0.05 (0.02)	0.04 (0.02)	0.67 (0.000)
20:3w6(DGLA)	3.14 (0.56)	2.98 (0.70)	0.75 (0.000)	2.99 (0.55)	2.79 (0.57)§	0.68 (0.000)
20:4w6 (AA)	7.8 (1.02)	8.0 (1.21)	0.82 (0.000)	8.75 (1.65)	8.4 (1.80)	0.79 (0.000)
Σ w6 FA	32.9 (1.53)	33.3 (1.84)	0.50 (0.006)	33.0 (1.06)	33.6 (1.02)§	0.29 (0.092)
						

18:3w3 (ALA)	0.32 (0.10)	0.32 (0.10)	0.67 (0.000)	0.15 (0.03)	0.17 (0.04)§	0.60 (0.000)
20:5w3 (EPA)	1.21 (0.34)	1.35(0.38)*	0.70 (0.000)	0.61 (0.25)	0.53 (0.32)	0.79 (0.000)
22:6w3 DHA	3.25 (0.88)	3.31 (1.09)	0.76 (0.000)	3.40 (0.73)	3.43 (0.97)	0.73 (0.000)
Σ w3 FA	4.8 (0.96)	5.0 (1.23)	0.68 (0.000)	4.2 (0.85)	4.1 (1.18)	0.73 (0.000)

Σ PUFA	37.9 (1.50)	38.5 (1.37)	0.57 (0.001)	37.4 (1.16)	37.8 (0.93)§	0.60 (0.000)

Ratios:						
AA/DHA	2.6 (0.86)	2.7 (1.12)	0.88 (0.000)	2.7 (0.62)	2.6 (0.58)§	0.83 (0.000)
w6/w3	7.1 (1.50)	7.13 (1.99)	0.58 (0.001)	8.3 (1.94)	8.9 (2.96)	0.71 (0.000)
						
						
						

In mothers of twins only one value is given. Mean (SD). For abbreviations see table 4.

Pearson correlation coefficients. Paired t-test was used for testing differences between 40 and 44 weeks, for mothers * p < 0.05 (n = 29) and for infants §p < 0.05, §§ p < 0.001 (n = 36).

### Longitudinal study of infants' plasma fatty acid levels up to 44 weeks

Comparisons between infants' early plasma samples and samples obtained corresponding to 40 and 44 weeks of gestational age showed strong significant increases of mean LA and ALA values compared with early samples. The ratio of these two variables did not change, while the total w6/w3 ratio increased slightly (p = 0.020, n = 30). The concentration of AA had decreased by 40 weeks (p < 0.0001, n = 30), and the negative correlation between LA and AA was strong (r = – 0.87, p < 0.0001, n = 36). In contrast to early infants' plasma also the correlation between ALA and DHA was significant (r = -0.39, p = 0.018, n = 36). At 44 weeks the correlation between LA and AA had strengthened (r = -0.94, p < 0.0001, n = 45), but that between ALA and DHA was no longer significant (p = 0.093). Therefore the increased intake of LA with age seemed to inhibit the AA synthesis, but a similar association with time was not found for ALA and DHA. For this time period, DHA was not significantly changed, although some initial high values had decreased markedly (Figure 2). The AA/DHA ratio had decreased (p < 0.0001, n = 30), supporting an inverse association between LA and AA. Mead acid and the EFA deficiency index were both lower at 40 weeks (p < 0.0001, n = 30) (Figure 3), and significantly lower than in the corresponding mothers' plasma (p < 0.0001). Also, the OA/LA ratio had decreased (p < 0.0001, n = 30).

Between 40 and 44 weeks LA and ALA increased further without changes to their ratio (Table 5). The concentrations of AA and DHA were similar. At 44 weeks, mead acid had decreased and no correlation was found to the main w6 or w3 FAs. The wide variations in the infants' early samples were in contrast to the much smaller variation at 40 and 44 weeks of gestational age (Figures 1 and 2). Comparison of the major w3 FAs in infants' and mothers' plasma phospholipids showed a similar decreasing pattern for DHA as for AA, but a markedly smaller variation in ALA compared with LA over time in the infants. The development of the w6/w3 ratio in mothers and infants with gestational age is shown in Tables 4 and 5. The EFA deficiency index (Figure 3) decreased in a similar pattern as mead acid (20:3w9), palmitoleic acid (16:1w7) and the OA/LA ratio (data not shown).

### The influence of breastfeeding on infants' and mothers' fatty acid pattern

**Figure 4:**
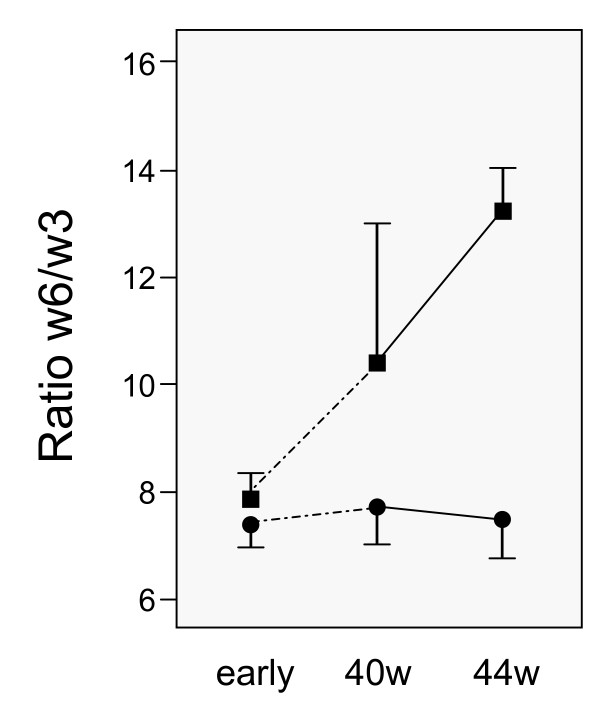
**The ratio of the sum of w6/w3 fatty acid concentrations (FAs) in infants' plasma phospholipids at 1 week of age (early) and at the gestational ages of 40 and 44 weeks**. Infants, whose mothers stopped breastfeeding during the observation period (black squares), and infants, whose mothers were still breastfeeding at 44 weeks (black circles) are indicated. Data are given as means with 95% confidence intervals.

**Figure 5:**
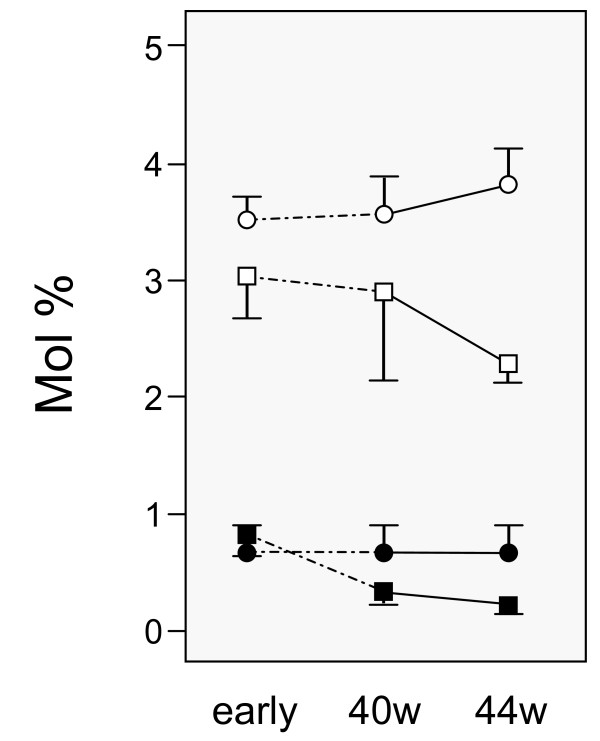
**Docosahexaenoic acid (DHA) (22:6w3, unfilled symbols) and eicosapentaenoic acid (20:5w3, filled symbols)concentrations in infants' plasma phospholipids at 1 week of age (early) and at the gestational ages of 40 and 44 weeks**.(squares) infants whose mothers stopped breastfeeding during the observation period, (circles) infants whose mothers were still breastfeeding at 44 weeks. Data are given as means with 95% confidence intervals.

The feeding of the infants during the first period of life significantly influenced their plasma phospholipid FA pattern. Plasma from infants who had been fed mothers' milk until up to 44 weeks' gestational age, group L ("long breastfeeding") (n = 38), were compared with infants who had stopped breastfeeding at any time before 44 weeks, group S ("short breastfeeding") (n = 7). The w6/w3 ratio in breast milk and in early infants' plasma samples did not differ between the two groups, the latter being 7.6 and 7.9 in groups L and S, respectively (p = 0.21). At 40 weeks the ratio differed significantly (p = 0.003) and it differed even more at 44 weeks (p < 0.0001) (Figure 4). In group L the DHA level in infants' plasma in fact increased between 1 week of age and 44 weeks of gestational age (p = 0.018) (Figure 5), and despite an increase in w6 FA, the w6/w3 ratio at 44 weeks had not changed. Contrary to this, the EPA and DHA plasma concentrations fell considerably in the infants of group S (p < 0.0001) (Figure 5), while ALA was equal in the two groups. Infants in group S also had a lower AA concentration (p < 0.0001), and a higher concentration of LA (p < 0.0001) than group L at the age corresponding to 44 weeks of gestation.

Mothers of infants in group L had decreasing DHA plasma concentrations, especially between 1 week postnatal and the expected time of birth (40 weeks), and no difference was found between mothers and infants at 44 weeks. At the same time the ALA concentration in mothers' plasma was approximately twice as high as that in the infants' plasma (p < 0.0001). The AA concentration was relatively constant over time, but significantly lower than in the infants (p = 0.006), while LA did not differ. In the mothers of group S, the plasma concentrations of DHA and w3 FAs showed a restitution between week 40 and week 44, and the w6/w3 ratio decreased during the same period of time. Corresponding to the gestational age of 44 weeks, DHA and w3 FAs were higher and the w6/w3 ratio was lower in mothers than in the infants of group S (p < 0.0001). Mead acid was higher in mothers' than in infants' plasma at 44 weeks (p < 0.0001) (Table 5). Both at 40 and at 44 weeks, mead acid had significant negative correlations to LA and DHA in mothers' plasma. In the mothers of group L, mead acid in plasma was predicted by the LA concentration (β = -0.74, p < 0.0001), and DHA (β = -0.69, p < 0.0001; R = 0.84, R^2 ^= 0.71, p < 0.0001, n = 32), indicating that these FAs explained 70% of the variance in the mead acid concentration.

Mothers of group L (n = 31) had a lower energy intake of fat (p = 0.02), including SFA (p = 0.04) and MUFA (p = 0.015), but a higher intake of carbohydrates (p = 0.02) than mothers of group S (n = 9). There were no differences between these groups for the P/S ratio or dietary intake of w6 and w3 FAs or the w6/w3 ratio in breast milk. The mothers of group L had a lower BMI (p = 0.007) and a higher educational status (p = 0.002) and more rarely smoked cigarettes (p = 0.01). There were no differences between the groups regarding gestational length, delivery, neonatal morbidity, sex, parity, birth weight, or size of the infants. The multiple logistic regression analysis indicated that smoking was the strongest predictor of shortness of breastfeeding (odds ratio, OR = 33.2, CI 11.0–100.3). The mothers' education also had a strong impact (lowest category OR = 20.4, CI 5.0–82.9, and intermediate education OR = 2.1, CI 0.64–7.0, compared with highest education, i.e. university). Furthermore, a higher BMI (OR = 1.6, CI 1.32–1.98) also independently predicted shorter breastfeeding.

## Discussion

This study presents a comprehensive prospective exploration of the associations between mothers' food intake, breast milk and plasma FA patterns early after birth up to the age corresponding to 44 weeks of gestation in mothers and their premature infants. The study forms the basis for further, ongoing longitudinal long-term follow-up studies. The initial large interindividual variation in EFAs in infants' plasma persisted during this early period of life although the pattern of increasing concentrations of LA and ALA and the relatively stable or decreasing concentrations of DHA and AA were clearly visible (Figures 1 and 2). The variability of FA concentrations in the mothers' plasma was much lower than in the infants for LA and AA, but not for ALA and DHA. Of special interest was the impact of breastfeeding on both infants' and mothers' plasma FA concentrations during the early postnatal period, indicating that the supply of LCPUFA-enriched formula milk cannot fully compensate for breastfeeding. This suggests that premature infants may be at special risk if not supplied with breast milk from their mothers (Figures 4 and 5).

### Impact of the mothers' diet

The mothers' diet had a relatively low energy content and, in a majority of the cases, a low content of EFAs both of the w6 and of the w3 series. It has been argued previously that low intake of w3 may contribute to premature birth [34], which would be supported by our study, since most mothers had w3 intake below recommended levels. It may be argued that the food composition did not mirror the usual food composition of the women, because the food intake records were done when most women were still in the hospital and under stress. However, when the dietary assessment was repeated 6 months later the results were not significantly different, except that the energy intake was 20% lower than at the first recording. This indicates that it is unlikely that the energy intake was underreported at the first investigation. The strong correlations between dietary factors and infants' FA concentrations suggest that more attention should be directed towards mothers' food intake during pregnancy. Dietary intake of both LA and ALA was strongly correlated to the total intake of fat (E %), but in breast milk only LA was associated with the mother's dietary intake, probably because ALA is considered to be oxidized to a greater extent [35]. This may be cause for concern since it has been suggested that at low fat and energy intake, more EFAs may be β-oxidized [36], which would further increase the risk that the infant may get an insufficient supply of EFAs in the breast milk. Mothers in this study, with the most "ideal" diet according to general recommendations (i.e. a low fat-high carbohydrate intake) also gave birth early to infants with the lowest LA concentrations. Our results suggest a risk that recommendations of low fat intake during pregnancy may negatively affect the supply of EFAs to the foetus and infant, which may not be possible to restore later in life and may have negative consequences for future health [37]. Linoleic acid as well as ALA in the breast milk was predicted by the P/S ratio of the mothers' dietary intake and to some extent by the PUFA level in the mothers' plasma. This confirms earlier observations that both diet and mobilization of fat reserves are important for breast milk composition [9].

### The impact of placental transfer compared with breast milk

As expected, LCPUFAs were found to predominate in the cord plasma. By comparison, the concentrations of AA and DHA were relatively low in mothers' milk, where LA and ALA dominated, which raises concerns about whether the large need of LCPUFAs, especially DHA, can always be satisfied by breastfeeding in prematurely born infants. Previous studies report a much higher concentration of LCPUFAs in breast milk [12,14]. The difference between cord blood and infants' early plasma samples indicated a much more efficient transfer of LCPUFAs via the placenta than through breast milk. The variation in early plasma samples from the infants was large for these more important LCPUFAs, with AA ranging from 8 to 19 mol% and DHA from 2.5 to 7 mol%. The concentrations were not associated with gestational age or birth weight and therefore probably reflected the different status of the mothers. A minor part may also be associated with different capacity in the newborns to transform EFAs to LCPUFAs [38-41].

### The impact of breastfeeding and mothers' fatty acid status

The concentrations of AA and DHA, but not of LA and ALA, showed a positive correlation between breast milk and infants' plasma. On the other hand, correlation between mothers' and infants' early plasma samples could only be found for ALA, EPA and DHA. This may be due to fast conversion of LA to AA. This changes with time, since at 40–44 weeks the high LA concentrations seemed to decrease the transformation to AA [35], as supported by the decreased AA/DHA ratio despite unchanged DHA concentrations. This interpretation was also supported by the findings in the group showing very low initial LA concentrations and significantly higher AA concentrations in infants' plasma. Interestingly, these infants differed in length, supporting earlier findings that AA is important for growth [42]. High concentrations of LA may inhibit the Δ6 desaturase activity and would thus explain the inverse association between LA and AA [43].

The DHA and EPA levels in mothers' plasma were closely correlated to these levels in both breast milk and infants' plasma, corroborating results from intervention studies by Innis [14]. Gestational age was another significant covariate, pointing to the importance of increasing mothers' intake and/or storage of w3 FAs with time [5,44]. The close correlations between mothers' plasma and breast milk obtained at the same time indicated less influence of the mammary gland on the FA pattern. In rats, mammary glands have been shown to contain Δ5 and Δ6 desaturases, which are stimulated by a diet low in w6 FAs [45], probably to uphold the synthesis of AA, indicating the biological importance of keeping the balance of the very important systems of prostanoid precursors (cf 44). The infants with very low LA concentration in plasma at 1 week of age had higher AA concentrations, which would support this feedback system, although we could not evaluate whether this was related to enzyme systems in the infants or in the mothers.

### Mead acid, an indicator of essential fatty acid deficiency

Although the mothers had a relatively low EFA intake, the plasma phospholipids pattern of FAs did not indicate a real deficiency. Nevertheless, mead acid in cord blood was very high and indicated a relative deficiency, probably associated with the demands of the foetus during pregnancy [5]. High concentrations of mead acid in pregnancy have previously been observed [46,47]. In our group of mothers these concentrations decreased rapidly after birth, but increased later again in those mothers who continued breastfeeding. They decreased faster and continuously in the infants and could hardly be detected after 40 weeks of gestational age. This may indicate that also in newborns mead acid is mainly associated with the w6 series of FAs, and that it is a sensitive marker of insufficient EFA supply also at this age. Mead acid has been proposed to be a substitute for AA in biological membranes because of similar biochemical structure, and therefore the mead acid/AA ratio has been used as an index of EFA deficiency. The often markedly increased mead acid in the early infants' plasma samples was associated with low plasma concentrations of LA and AA, but multiple regression analyses have indicated that mead acid was also associated with w3 FA concentrations. It is tempting to speculate whether the high concentration of mead acid in cord blood and in infants' early plasma samples may indicate a special function of this FA during foetal life.

### The impact of breastfeeding up to 40–44 weeks gestational age

Breastfeeding mothers offer compensatory protection to their premature infants through the breast milk, especially concerning DHA content in the short time up to 44 weeks of gestational age. The major group of mothers who were breastfeeding to 44 weeks (group L) showed successively decreasing plasma levels of DHA, while their infants had significantly increased their plasma level at 44 weeks compared with their initial values (Figure 5). The decreasing DHA concentrations in the lactating mothers may support the observation by Al et al. [48], that mothers are at risk for subsequent DHA depletion by many pregnancies. Also, the concentration of AA in group L at 44 weeks was higher in the infants than in their mothers, indicating that both these LCPUFAs were selectively enriched in infants' plasma. In the small group of mothers with short breastfeeding (group S), DHA levels increased between 40 and 44 weeks, and the w6/w3 ratio decreased, indicating that DHA was retained. Correspondingly, in the infants of this group a marked fall of both DHA and AA was seen in plasma, despite the fact that the infants were supplied with LCPUFA-enriched breast milk substitutes. The influence of other risk factors identified in this group of mothers must therefore be considered, especially smoking, which was more common in the mothers with short breastfeeding. Since maternal smoking has been shown to impair DHA synthesis [49], insignificantly lower DHA levels already in the early plasma samples in the group of smoking mothers may support such association. These observations further stress the need for supplementation with LCPUFA during pregnancy [50].

Between 40 and 44 weeks of gestational age, the DGLA and AA concentrations in the infants were already similar to those in the mothers, but the w3 FAs, especially ALA and EPA, were still lower while the DHA concentration was similar. Since others have shown that the LCPUFA content decreases from birth to mature milk in both term and preterm infants [12,51], the results may indicate that newborn premature infants have a fairly good capacity to synthesize LCPUFA [38].

The steadily increasing w6/w3 ratio in infants not breastfed up to week 44 was probably related to the higher w6/w3 ratio in formula, which has been recommended to range from 8 to 9[52], compared with breast milk which in our study averaged 5.6. It was obvious that formula feeding influenced the FA pattern in infants, with a rapidly increasing w6/w3 ratio and decreasing AA and DHA concentrations. A special concern is that mothers have been warned not to eat fish due to environmental pollution. Such warning may result in a too low fish intake, as indicated by the food records and also described by others [50].

*In summary*, this study shows that a majority of unselected mothers of late premature infants in an ordinary urban setting in Sweden had a low energy and PUFA intake, and especially a low intake of w3 FAs, which was reflected in a wide range of w3 FA concentrations in infants' early plasma samples. Some infants born to mothers who reported having strictly kept to the nutritional recommendations, had extremely low LA concentrations at an early age. Infants of mothers, who continued breastfeeding up to the age corresponding to 44 weeks of gestation, kept their w3 FA concentrations in plasma, but those who did not fully breastfeed had rapidly decreasing concentrations and, simultaneously, markedly increased w6/w3 FA ratios in their plasma. Our study indicates that EFAs, and especially w3 FAs, may be unsatisfactorily supplied to pregnant mothers. In the context of the importance of satisfactory supplementation of LCPUFAs during this early period of life, controlled randomized intervention studies, starting early in pregnancy with long-term follow-up, are urgently needed.

## Competing interests

None of the authors had a personal or financial conflict of interest. BS had in an Obesity trial study financial support from Equazen LTD, London, Great Britain, and iQmedical AB, Sweden.

## Authors' contributions

K-GS and EB cared for the patients and participated in the planning of the study, the calculations and the writing the manuscript. CL-P participated in the planning of the study and the writing the manuscript. MP was responsible for the statistical analysis and BS was responsible for the lipid laboratory tests, for calculations and for writing the manuscript. All authors have approved the final manuscript.
